# Impact of Early Life Adversity on Reward Processing in Young Adults: EEG-fMRI Results from a Prospective Study over 25 Years

**DOI:** 10.1371/journal.pone.0104185

**Published:** 2014-08-13

**Authors:** Regina Boecker, Nathalie E. Holz, Arlette F. Buchmann, Dorothea Blomeyer, Michael M. Plichta, Isabella Wolf, Sarah Baumeister, Andreas Meyer-Lindenberg, Tobias Banaschewski, Daniel Brandeis, Manfred Laucht

**Affiliations:** 1 Department of Child and Adolescent Psychiatry and Psychotherapy, CIMH Medical Faculty Mannheim/Heidelberg University, Mannheim, Germany; 2 Department of Psychiatry and Psychotherapy, CIMH Medical Faculty Mannheim/Heidelberg University, Mannheim, Germany; 3 Department of Neuroimaging, CIMH Medical Faculty Mannheim/Heidelberg University, Mannheim, Germany; 4 Department of Child and Adolescent Psychiatry, University of Zurich, Zurich, Germany; 5 Center for Integrative Human Physiology, University of Zurich, Zurich, Germany; 6 Neuroscience Center Zurich, University of Zurich and ETH Zurich, Zurich, Germany; 7 Department of Psychology, University of Potsdam, Potsdam, Germany; University Medical Center Goettingen, Germany

## Abstract

Several lines of evidence have implicated the mesolimbic dopamine reward pathway in altered brain function resulting from exposure to early adversity. The present study examined the impact of early life adversity on different stages of neuronal reward processing later in life and their association with a related behavioral phenotype, i.e. attention deficit/hyperactivity disorder (ADHD). 162 healthy young adults (mean age = 24.4 years; 58% female) from an epidemiological cohort study followed since birth participated in a simultaneous EEG-fMRI study using a monetary incentive delay task. Early life adversity according to an early family adversity index (EFA) and lifetime ADHD symptoms were assessed using standardized parent interviews conducted at the offspring's age of 3 months and between 2 and 15 years, respectively. fMRI region-of-interest analysis revealed a significant effect of EFA during reward anticipation in reward-related areas (i.e. ventral striatum, putamen, thalamus), indicating decreased activation when EFA increased. EEG analysis demonstrated a similar effect for the contingent negative variation (CNV), with the CNV decreasing with the level of EFA. In contrast, during reward delivery, activation of the bilateral insula, right pallidum and bilateral putamen increased with EFA. There was a significant association of lifetime ADHD symptoms with lower activation in the left ventral striatum during reward anticipation and higher activation in the right insula during reward delivery. The present findings indicate a differential long-term impact of early life adversity on reward processing, implicating hyporesponsiveness during reward anticipation and hyperresponsiveness when receiving a reward. Moreover, a similar activation pattern related to lifetime ADHD suggests that the impact of early life stress on ADHD may possibly be mediated by a dysfunctional reward pathway.

## Introduction

Accumulating evidence suggests that adversity in early childhood may impair human brain development and mental health later in life [1]–[3]. Moreover, clinical studies have highlighted striking effects of early life adversity on the development and persistence of mental disorders such as attention deficit/hyperactivity disorder (ADHD) [4]–[7]. Among the mechanisms mediating the detrimental impact of early adversity on psychopathology and brain development, alterations of the mesolimbic reward pathway have been suggested to play a major role [8]–[10]. Several functionally related brain regions have been implicated in the processing of rewards by a large body of functional magnetic resonance imaging (fMRI) findings and have been related to different stages of reward processing [11], [12]. These findings emphasize a functional dissection of reward processing. While anticipation or “wanting” of a reward addresses the motivational aspect to receive a reward, reward delivery or “liking” has been interpreted as the hedonic impact of a reward producing the feeling of pleasure [13].

Common regions that are preferentially activated during the anticipation of rewards encompass the ventral striatum (VS), including the nucleus accumbens, ventral caudate nucleus and ventral putamen. Another region suggested to be involved in the delivery of rewards covers the medial orbitofrontal cortex, adjacent parts of the ventromedial prefrontal cortex, medial and dorsal caudate as well as putamen. With regard to ADHD, most studies have demonstrated a reduced activation of the VS during reward anticipation in patients compared to healthy controls [8], [9], [14]–[16], while for the delivery phase, an increased activation of the caudate nucleus was observed [15]–[17]. These effects are in line with the dopamine transfer deficit model, which postulates a diminished dopaminergic response shift from the actual reward to the anticipatory stimulus, but a remaining strong response during reward delivery [18], [19].

In contrast to neuroimaging research, fewer studies have examined electrophysiological correlates of anticipatory reward processing. One anticipatory event-related potential (ERP) which has been investigated more systematically measuring an electroencephalogram (EEG) is the contingent negative variation (CNV) type activity, a slow negative potential shift before target or feedback stimuli, with a maximum over central sites, elicited by preparation and anticipation paradigms [20], [21]. If feedback immediately follows the response, the CNV reflects reward anticipation along with motor and cognitive preparation or time estimation. If feedback is delayed, reward anticipation is also postponed and is mainly reflected by the feedback or stimulus-preceding negativity (SPN) following the CNV and the motor response [22]. So far, some findings have indicated higher CNV-like activity during reward anticipation [23]–[25], although other studies did not find an effect of reward anticipation on the target-preceding CNV in tests in which feedback was postponed or predictable [26], [27]. In turn, several studies have shown a reduced CNV for children with ADHD or adults with a childhood diagnosis of ADHD, acting on a cued continuous performance test (CPT), investigating developmental effects of impaired cognitive brain functions [28]–[31].

Increasing evidence has implicated the neural circuitry of reward in altered brain function resulting from exposure to early life adversity. At the behavioral level, impaired responding to rewarding stimuli in maltreated individuals was reported [32]. These individuals exhibited faster reactions for risky options in a decision-making task than controls, but lacked the typical increase in response speed with the chance of winning. Further evidence for a reduced sensitivity to reward was provided in an fMRI study [10]. Young adults maltreated during childhood showed a blunted basal ganglia response (left putamen, left globus pallidus) and less positive ratings of reward cues during reward anticipation. Another study underscored these results by demonstrating a decreased activation in the VS to reward-predicting cues in Romanian adoptees who had experienced global early deprivation [33]. In these studies, no effect of early adversity on reward delivery was observed, suggesting that adversity might specifically affect responses to reward-predicting cues. However, a recent study by Kumar et al. [34] investigating the impact of acute stress found differential effects on phases of reward processing, with increased neuronal activation in the caudate and the amygdala during reward anticipation and decreased activation in the caudate and the putamen while receiving a reward. Hence, acute and early chronic stress seem to impact on the anticipatory and delivery stage of reward processing in specific ways, most likely mediated by alterations of the hypothalamus-pituitary-adrenal (HPA) axis [35].

In the present study, the impact of early adversity on reward processing was examined in a large sample of young adults from an epidemiological cohort study followed since birth. Using a monetary incentive delay (MID) task offering either money or verbal feedback, simultaneous EEG-fMRI was recorded in order to detect alterations at different stages of reward processing. Given the fact that the verbal feedback (control condition) of the MID represents a special reward characteristic, such as if receiving a social reward [36], [37], modality-specific differences in rewarding quality will be examined. The use of EEG and fMRI provides both high spatial and temporal resolution of neuronal alterations during reward processing. Especially, the EEG enables a cue related analysis of time-resolved neurophysiological signatures within the anticipation phase as recently demonstrated by Plichta et al. [38]. First, we hypothesized that activation of reward-related areas induced by the anticipation of a monetary reward, especially the VS, would decrease with the level of early adversity. Second, we expected the same effect for the EEG, i.e. that the CNV, reflecting the motivational signature of reward anticipation, would decrease with increasing adversity. Third, in line with previous research, no adversity-specific alterations of the neuronal response to monetary reward outcome were predicted [10], [33]. Fourth, we hypothesized that reward-related neuronal activation was related to lifetime ADHD symptoms, showing decreasing neuronal activity during reward anticipation and increasing activation during reward delivery with the level of ADHD [14], [15], [17].

## Materials and Methods

### Ethics statement

The current assessment was approved by the ethics committee of the University of Heidelberg. After complete description of the study to the participants, written informed consent was obtained. For assessments during childhood (age three months to 15 years) written informed consent was obtained from parents on behalf of the children.

### Sample

This investigation was conducted in the framework of an epidemiological cohort study examining the long-term outcome of early risk factors from birth into adulthood. Detailed information about this study has been published elsewhere [39], [40]. The initial sample consisted of 384 infants born between 1986 and 1988 of predominantly (>99%) European descent, who were consecutively recruited from two obstetric and six children's hospitals of the Rhine-Neckar Region of Germany. Only firstborn children with singleton births and German-speaking parents were enrolled in the study. Assessments were first conducted at the age of three months and subsequently at regular intervals throughout development, most recently in young adulthood. From the initial sample, 18 (4.7%) were excluded due to severe handicaps and 57 (14.8%) were dropouts, leaving a final sample of 309 for the current assessment. From these, 122 individuals had to be excluded due to usual contraindications for MRI and EEG, current psychopathology or psychotropic medication. This sample of N = 187 individuals participated in a simultaneous EEG-fMRI measurement. Another twenty-five participants were discarded due to movement artifacts (>3 mm) or insufficient EEG quality, leaving a final sample of N = 162 participants (mean age = 24.4 years; 58% female).

### Psychological assessment

Early adversity was assessed using a standardized parent interview according to an ‘enriched’ family adversity index as proposed by Rutter and Quinton [41]. The interview comprised 11 items covering characteristics of the family environment (e.g., Overcrowding: More than 1.0 person per room or size of housing ≤50 m^2^), the parents (e.g., Parental psychiatric disorder: Moderate to severe disorder according to DSM-III-R criteria) and their partnership (e.g., Unwanted pregnancy: An abortion was seriously considered) during a period of one year prior to the assessment (see Table S1). A total early family adversity (EFA) score was formed by counting the number of items present at the 3-month assessment [mean = 1.71±1.87; range: 0–7; Cronbach's alpha = .72]. The EFA index is a prospective and comprehensive measure of family adversity and does not exclusively focus on emotional and sexual abuse or neglect. Empirical evidence has largely confirmed the cumulative risk hypothesis that the likelihood of unfavorable child outcomes increases with the number of adversity factors [42]. Furthermore, a series of studies conducted in the context of the Mannheim Study of Children at Risk have provided evidence of the current validity of the family adversity measure [39], [43], [44]. The Structured Clinical Interview for DSM-IV (SCID-I German version) [45] was administered to measure young adults' psychiatric disorders. To examine current drug use, participants completed a substance use inventory [46]. Lifetime ADHD symptoms were assessed with the Mannheim Parent Interview (MEI) [47] at age 2, 4, 8 and 11 years and with the Schedule for Affective Disorders and Schizophrenia for School- Aged Children (K-SADS-PL) [48], German version [49] at age 15 years and sum scores were formed, indexing the severity and persistence of ADHD. The MEI is a highly structured interview adapted from Rutter's parent interviews to include all symptoms related to major DSM-IV diagnoses, and has been shown to be a sensitive measure of child disturbance [40], [50], [51]. With regard to ADHD, agreement with an independent child psychiatric examination was seen in 100% of cases. The K-SADS is a widely used structured diagnostic interview completed independently by parents and adolescents with established reliability and validity [52]. Information from different sources was combined by the logical operator OR.

### Experimental paradigm

**Figure 1:**
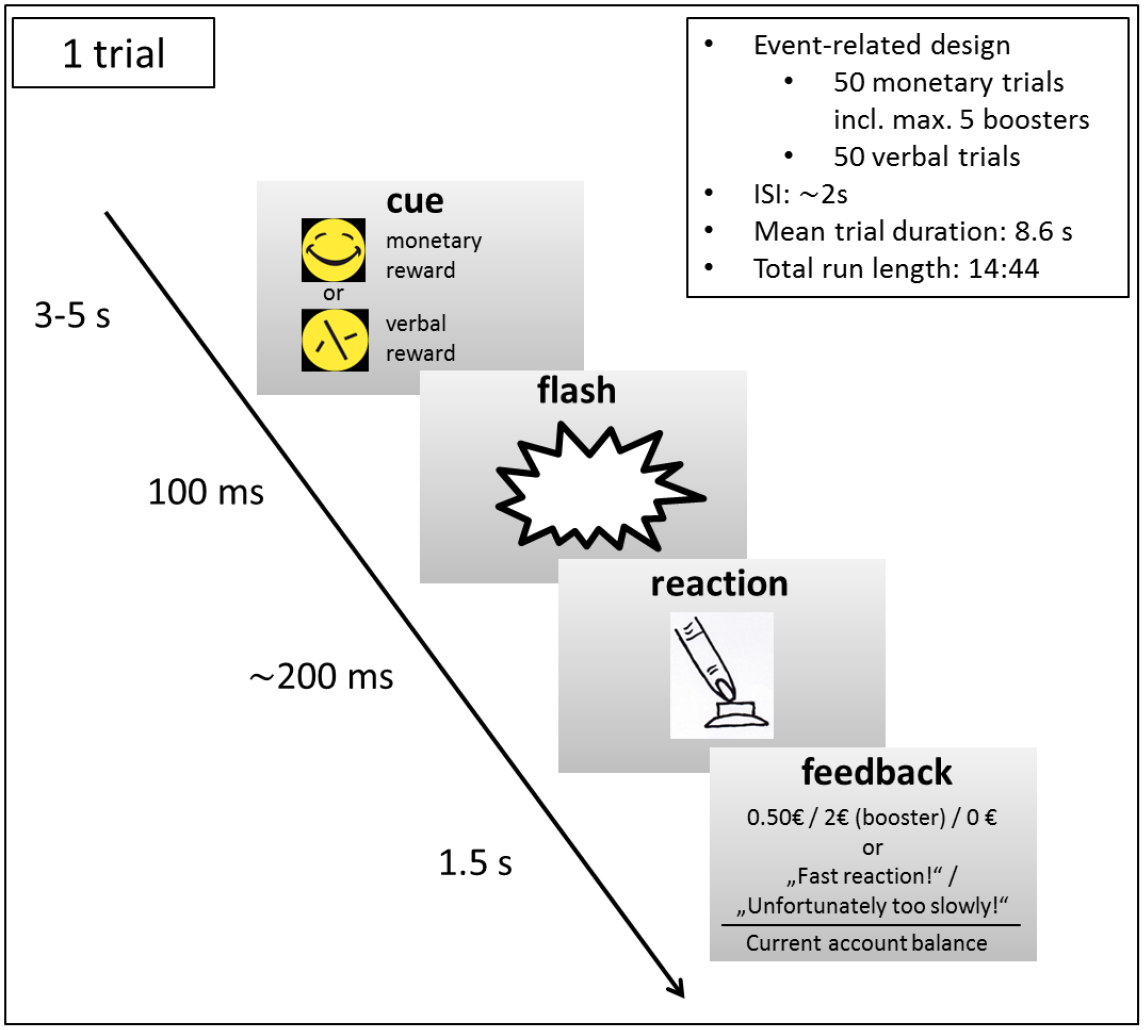
MID paradigm. The task requires a fast button press after a flash, indicated by either a laughing or a scrambled smiley, to receive either monetary or verbal feedback.

The fMRI paradigm (Figure 1) was a modified version of the MID task [53], [54], probing reward anticipation and delivery which was adapted to simultaneous EEG-fMRI measurements. Previous results using this paradigm have shown reliable and robust activation of the VS [55]. The task requires a fast button press directly after a flash target following a reward anticipation cue to win a potential reward. Targets followed cues which consistently signaled different types of reward anticipation (unlike in reversal-learning paradigm): either a happy smiley signaling that responding fast enough would yield a monetary feedback (0.50 Euro), or a scrambled smiley indicating only verbal feedback (“Fast reaction!”; usually treated as the control condition). Smileys were used to further minimize learning effects. After every trial, the participants were informed about the current account balance. Boost trials with a monetary reward of 2 Euro instead of 0.50 Euro were given approximately every eighth win trial in order to improve the participants' motivational level. In total, 50 monetary and 50 verbal trials were presented in a pseudo-randomized order. The cue duration (and consequently trial length) was jittered (3-5sec) to cover the whole hemodynamic response function (HRF). The reaction time window (common for both reward conditions) was adaptively tailored to account for inter-individual differences and to yield comparable winnings across participants.

### Data acquisition

**1. EEG.** The EEG was recorded inside the scanner using an MRI-compatible EEG system with 5 kHz sampling rate, 32 mV input range and 0.1–250 Hz band-pass filters. The signal was measured by equidistantly spaced silver/silver chloride (Ag/AgCl) scalp electrodes using EEG caps with twisted and fixed electrode cables (Easycap, Munich, Germany). The 60-channel EEG montage included most 10–10 system positions (for further information see Plichta et al. [55]). F1 served as recording reference, and F2 as the ground electrode. Four additional electrodes were placed to record the electrooculogram (EOG) and the electrocardiogram (ECG). Via optic fibers, the signal was transmitted from two MRI-compatible amplifiers (Brain Products, Gilching, Germany) outside the scanner room. Electrode impedances were kept below 20 kΩ, except for ECG and EOG electrodes (<30 kΩ) as well as reference and ground (<10 kΩ). The EEG was monitored while scanning using online correction software (RecView Brain Products, Gilching, Germany).
**2. fMRI.** The fMRI data was recorded on a 3 Tesla whole-body scanner (Magnetom Trio, Siemens Erlangen, Germany). fMRI data were measured using a T2*-weighted EPI sequence with the following parameters: 400 volumes, 36 slices in ascending order and oriented 20° steeper than AC-PC-plane, 3mm slice thickness, TR/TE = 2210/28 ms, FOV = 220×220 mm, 64×64 matrix, Flip angle = 90°. A T1-weighted anatomical 3D sequence (MPRAGE) was acquired for each subject. The paradigm was created with Presentation software (Neurobehavioural Systems Inc., Albany, USA) and presented via video goggles (Resonance Technology Inc., Northridge, USA). Performance of participants was recorded using response pads (Current Designs, Philadelphia, USA & Presentation software).

### Data analysis

**1. Behavior.** Reaction times (RT) were averaged across trials per condition (monetary, verbal) and the amount of win trials per condition was summed up. Condition differences were examined by means of paired t-tests, effects of EFA with linear regression analysis. The interaction between EFA and condition with regard to behavioral measures was obtained using repeated measures ANOVAs in SPSS Software package (Version 20, IBM Corp., Armonk, USA).
**2. EEG.** EEG data were corrected for MRI gradient [56] and cardioballistic artifacts [57] using standard template subtraction procedures as implemented in the Brain Vision Analyzer software 2.0 (Brain Products, Gilching, Germany). EEG data were digitally low-pass filtered (70 Hz) and down-sampled to 500 Hz. After exclusion of physical artifacts via raw data inspection, infomax independent component analysis (ICA) [6], [58] was used to remove ocular (blinks, movements) and residual cardioballistic artifacts [59] related to gradient modulation. EEG data were re-referenced to the average reference, baseline-corrected to a 500 ms pre-stimulus interval and low-pass filtered with a cut-off of 30 Hz. Segmentation into ERP epochs of 3.5 seconds began 500 ms prior to cue onset. ERP averages for both conditions (monetary and verbal feedback) were calculated for each participant. The CNV at electrode Cz, commonly showing the highest amplitude and therefore the best signal-to-noise [29], [38], was measured as the mean amplitude for the 2–3-second time window following cue onset.
**3. fMRI.** The fMRI data was analyzed using statistical parametric mapping (SPM8; Wellcome Trust Centre for Neuroimaging, London, UK). Preprocessing included slice-time correction, realignment (motion correction), spatial normalization into Montreal Neurological Institute space, resampling to 2×2×2 mm and spatial smoothing with an 8-mm full-width at half maximum (FWHM) Gaussian kernel. Spatial normalization was performed by coregistering the realigned mean image to the anatomical image, normalizing the anatomical image to the T1 template and applying these transformation parameters to the time series.

Individual first-level analysis was performed by linear regression analysis. A general linear model with eight regressors of interest (laughing and scrambled smiley, flash, response, monetary and verbal, win and no-win trials, respectively) was designed and convolved with the SPM hemodynamic response function (HRF). Six motion parameters were included in the design matrix and modeled as regressors of no interest. A high-pass filter with a cut-off frequency of 1/128 Hz was used to attenuate low-frequency components. All analyses were corrected for serially correlated errors by fitting a first-order autoregressive process (AR[1]) to the error term.

First-level contrasts were implemented in the second-level group analysis (monetary>verbal cue; win>no-win; monetary win>monetary no-win; verbal win>verbal no-win) with EFA embedded as a covariate of interest and controlling for gender. In a subsequent analysis, results were controlled for subclinical psychopathology as measured using the Young Adult Self-Report (YASR) [60] at the current assessment. A statistical threshold of p<.001 (uncorrected) was applied in a whole-brain analysis and, a region of interest (ROI) analysis was performed. ROIs were anatomically labeled with WFU PickAtlas [61], determining putamen, pallidum, ACC, thalamus, insula, hippocampus and substantia nigra as ROIs of interest [11], [12]. Results were thresholded at p<.05; family-wise error (FWE) corrected. The VS mask was defined according to Plichta et al. [38] as a fusion of the “caudate head” mask taken from the WFU-PickAtlas (human-atlas TD Brodmann areas+) and the “accumbens” mask from the Harvard–Oxford Subcortical Structural Atlas (implemented in FSLView 3.1.8; see http://www.cma.mgh.harvard.edu/fsl_atlas.html; probability threshold was set to 50%). The left and right VS were treated as separate ROIs. The hippocampus mask was divided along the y-axis in an anterior and a posterior part with MARINA [62] according to Poppenk et al. [63]. Mean beta values (across all voxels within ROIs) were imported into SPSS 20 for linear regression analysis. Post-hoc repeated measures ANOVAs, with *Phase* (Anticipation/Delivery) as the repeated factor and EFA as a covariate of interest were calculated for the left VS, right insula and left putamen ROIs. The same type of ANOVA was also conducted using the input of two different ROIs (left VS for reward anticipation/right insula for reward delivery), in order to directly examine the effects of phase in those regions which showed the highest activation in the separate ROI-analysis from each phase. In addition, a post-hoc factorial analysis provided a direct test of the interaction between condition (monetary vs. verbal) and outcome (win vs. no-win) during the delivery phase.

**4. Association between early life stress, ADHD symptoms and neuronal activation.** Pearson correlations were computed to establish the relationship of the significant cluster of neuronal activation with EFA and ADHD symptoms. A mediation analysis was conducted to assess whether a possible effect of EFA on ADHD symptoms is mediated by neuronal activation. Mediation was tested following previous work of our group [38] by means of the Sobel test [64] accompanied by a bootstrapping method with N = 5000 samples [65] using SPSS 20.

## Results

### Behavior

Behavioral data analysis showed a performance advantage for RT and number of win trials when contrasting the monetary with the verbal condition. Participants responded faster after the presentation of a monetary relative to a verbal cue (monetary: 195.81±26.78 ms; verbal: 225.52±41.96 ms; t_(161)_ = −10.56; p<.001) and won a monetary trial more often than a verbal trial (monetary: 28.55±2.97; verbal: 21.52±3.19; t_(161)_ = 15.01; p<.001). A significant effect of EFA on RT of monetary trials emerged (F_(1,160)_ = 9.22, p = .003), with RT increasing with the level of adversity. A similar effect was observed for the verbal reward condition (F_(1,160)_ = 6.45, p = .012), after exclusion of one participant who exceeded the 3-fold interquartile range. No effect of EFA on the number of win trials (monetary: F_(1,160)_ = .03; p = .863; verbal: F_(1,160)_ = .395; p = .531) and no interaction between EFA and condition on RT (F_(7,154)_ = .609, p = .748) or number of win trials (F_(7,153)_ = .076, p = .999) was found. All participants gained money (mean: 21.91 €±2.02; range: 16.00–26.50 €). No effect of EFA on payoff emerged (F_(1,160)_ = .040; p = .843).

### EEG

**Figure 2:**
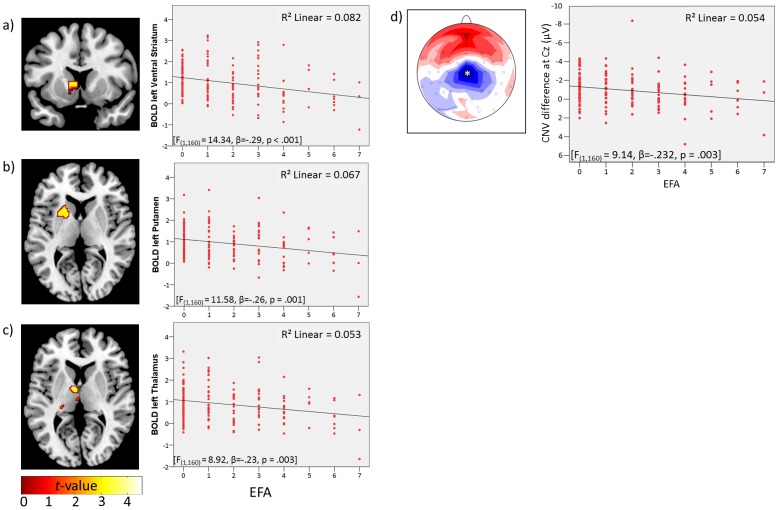
Left: neuronal activity (p_FWE_<.05; ROI corr.) for the contrast monetary > verbal reward during reward anticipation by early family adversity (EFA) in **a**) left VS, **b**) left putamen and **c**) left thalamus; right: scatterplots of the correlations between the mean BOLD response of the respective regions and EFA; **d**) left: Scalp distribution of CNV difference (monetary > verbal condition; mean difference: 2–3 sec after stimulus presentation) dependent on EFA; right: scatterplot of the correlation between CNV difference at Cz (marked with an asterisk) and EFA [F_(1,160)_ = 9.14, p = .003].

A task effect on the contingent negative variation (CNV) revealed that the anticipation of a monetary reward induced a higher CNV than the anticipation of a verbal reward [t_(161)_ = −7.18; p<.001; see Figure S3]. Furthermore, an effect of EFA indicated that the CNV (contrasting monetary to verbal reward) decreased when EFA increased (see Figure 2d).

### fMRI

**Figure 3:**
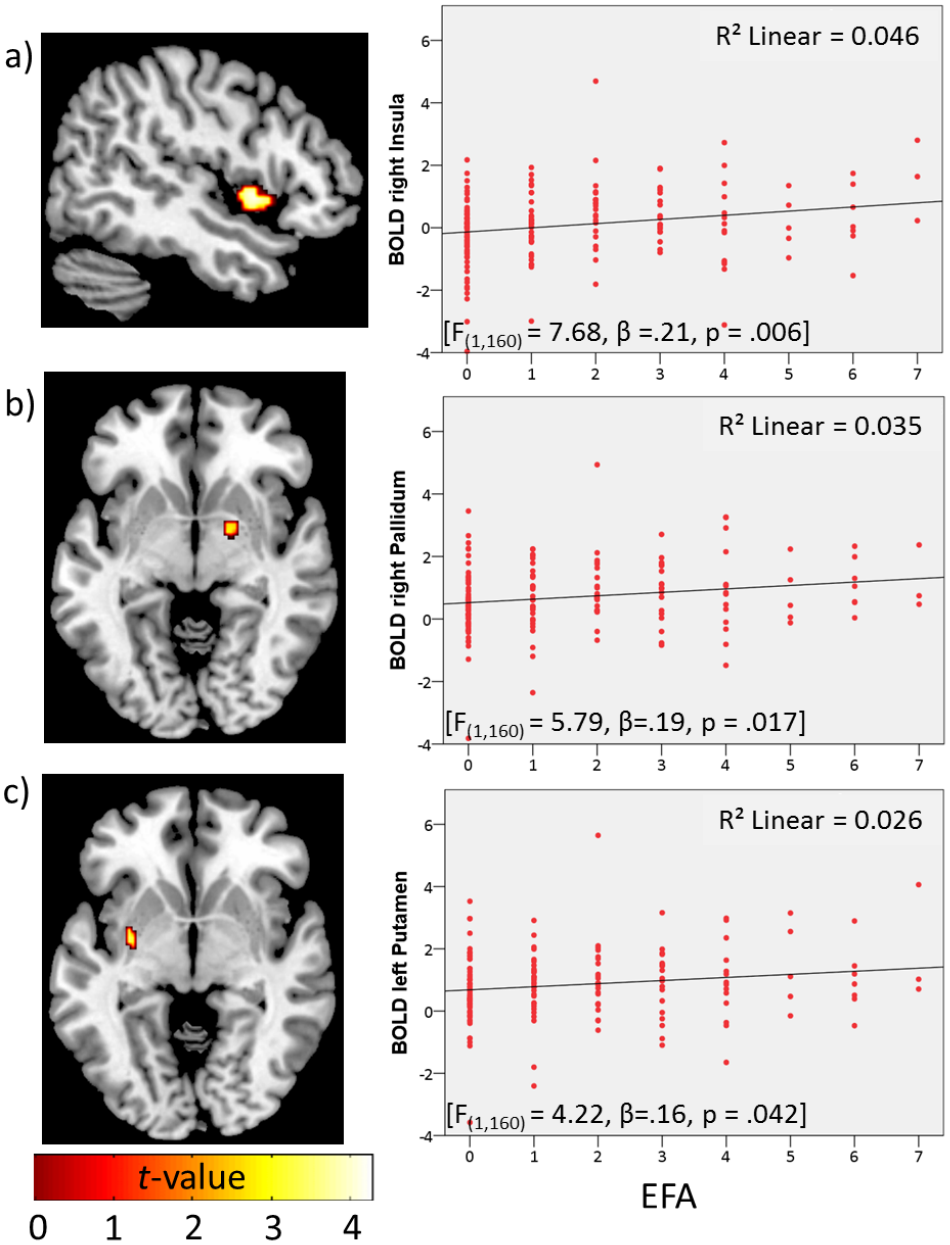
Left: neuronal activity (p_FWE_<.05; ROI corr.) for the contrast win > no-win trials during reward delivery by EFA in **a**) right insula, **b**) right pallidum and **c**) left putamen; right: scatterplots of the correlations between the mean BOLD response of the respective regions and EFA.

**Figure 4:**
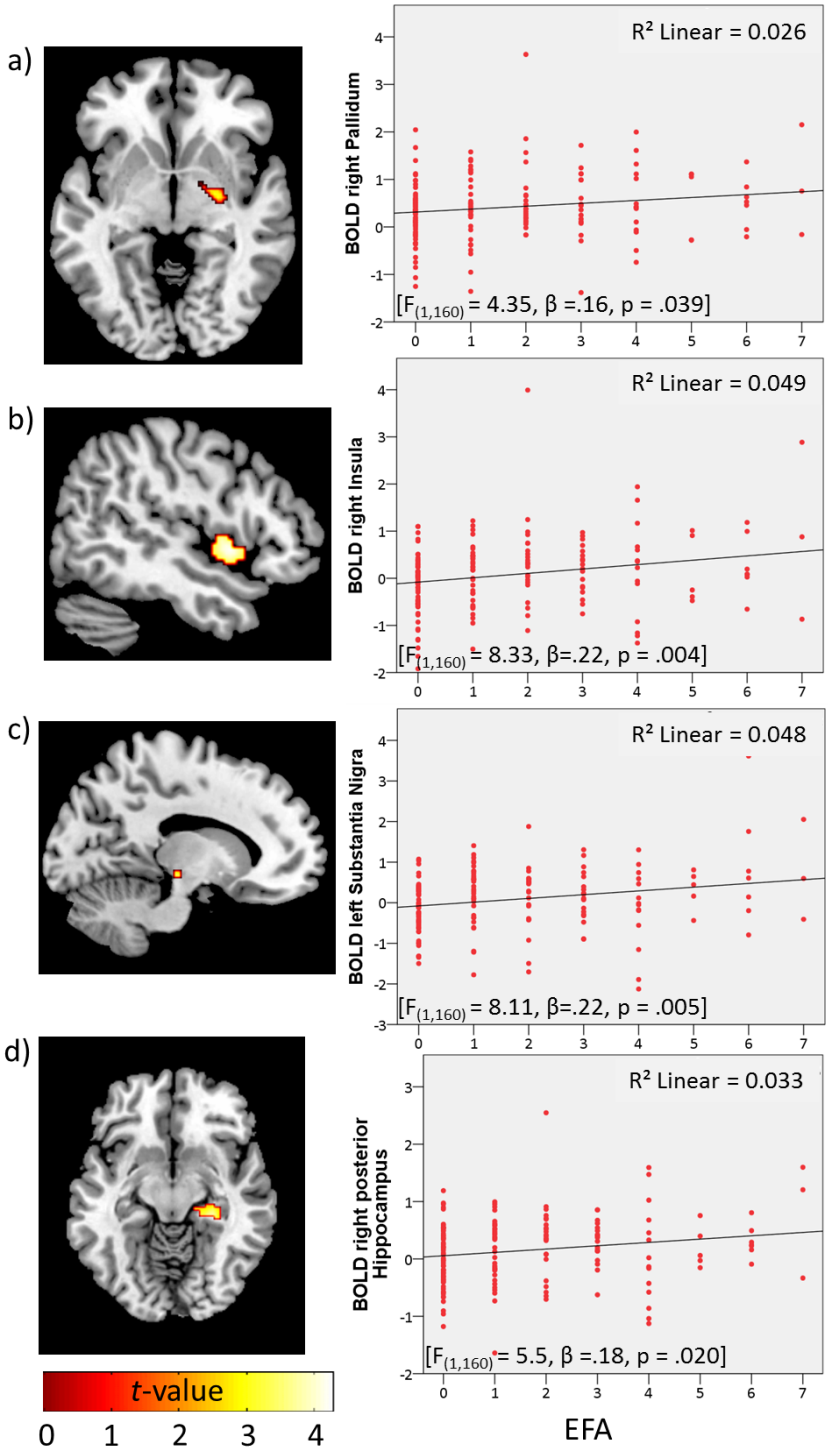
Left: neuronal activity (p_FWE_<.05; ROI corr.) for the contrast verbal win > no-win trials during reward delivery by EFA in **a**) right pallidum, **b**) right insula, **c**) left substantia nigra and **d**) right posterior hippocampus; right: scatterplots of the correlations between the mean BOLD response of the respective regions and EFA.

**1. Effects on reward anticipation.** Whole-brain analysis contrasting monetary to verbal reward anticipation revealed higher activation of reward-related regions (VS, supplementary motor area and anterior cingulate cortex, all p_FWE_<.0001; see Figure S1a). Furthermore, a ROI-analysis revealed a significant effect of EFA on the contrast (monetary>verbal) in the VS, putamen, pallidum, left thalamus, left insula, left ACC and right anterior hippocampus (p_FWE_<.05; ROI-corrected; see Table 1), indicating that activation in these regions decreased with the level of EFA. Figure 2a–c shows the respective activation maps together with corresponding extracted mean betas for the predefined ROIs, estimated by linear regression. Results remained constant or even improved (VS left: t = 4.65, p<.001; putamen left: t = 4.36, p = .001; pallidum left: t = 4.37, p<.001), when controlling for subclinical psychopathology at the current assessment.
**2. Effects on reward delivery.** Whole-brain analysis of the reward delivery phase induced similarly robust activation of reward-related areas (for the win>no-win contrast; task effect, pooling of monetary and verbal feedback), specifically the putamen, caudate, left inferior frontal gyrus, and right dorsolateral prefrontal cortex (p_FWE_<.0001; see Figure S1b). In addition, a ROI-analysis indicated a significant effect of EFA on the former mentioned contrast, with increasing activation in the bilateral insula, right pallidum and bilateral putamen with the level of EFA (p_FWE_<.05; ROI-corrected; see Table 2). Activation maps and extracted mean betas are displayed in Figure 3. Separate analysis for verbal outcome revealed that with increasing EFA, participants showed higher activation in the bilateral insula, pallidum, substantia nigra and right posterior hippocampus (contrasting verbal win>no-win; p_FWE_<.05; ROI-corrected; see Table 3). Activation maps and extracted mean betas are displayed in Figure 4. In contrast, there was no significant EFA effect on activation in reward-related areas for monetary outcomes (regression analysis for left insula: F_(1,160)_ = 1.41, p = .24; right insula: F_(1,160)_ = 1.47, p = .23; left putamen: F_(1,160)_ = 1.42, p = .24; right putamen: F_(1,160)_ = 2.7, p = .10; right pallidum: F_(1,160)_ = 2.73, p = .10). Results for both contrasts (win>no-win, verbal win>no-win) were attenuated, when controlling for subclinical psychopathology at the current assessment, but still remained significant (win>no-win: putamen right: t = 3.56, p = .021, pallidum right: t = 3.19, p = .021, insula right: t = 4.17, p = .005; verbal win>no-win: pallidum right: t = 3.43, p = .011, posterior hippocampus right: t = 3.97, p = .003, insula right: t = 4.15, p = .006).The factorial interaction (directly testing increased EFA-related modulation of verbal>monetary wins) revealed overlapping activation with the verbal outcome in a hippocampal area, indicating that the difference between the EFA-related activation for win>no-win trials was higher for verbal than for monetary outcome.

**Table 1:** Regional BOLD changes to monetary > verbal reward by EFA.

	MNI	k	t	p
Region	coordinates	cluster size	value	value*
VS left	−8 4 4	157	4.52	.000
VS right	14 14 6	49	3.18	.019
Putamen left	−18 6 12	434	4.10	.003
Putamen right	34 8 −2	394	3.75	.010
Pallidum left	−10 2 2	180	4.30	.006
Pallidum right	18 4 6	174	3.62	.006
ACC left	−14 36 20	110	4.24	.003
Thalamus left	−10 −2 6	44	4.13	.018
Insula left	−38 18 −6	41	3.81	.007
Anterior hippocampus right	18 −6 −12	15	3.56	.010

* FWE corrected at a threshold of .05, k≥10.

**Table 2:** Regional BOLD changes to win > no-win trials by EFA.

	MNI	k	t-	p
Region	coordinates	cluster size	value	value*
Insula right	48 8 −2	78	4.25	.004
Insula left	−46 4 −4	13	3.84	.016
Putamen left	−32 −4 4	17	3.77	.011
Putamen right	32 2 4	24	3.63	.017
Pallidum right	18 0 −4	23	3.31	.015

* FWE corrected at a threshold of .05, k≥10.

**Table 3:** Regional BOLD changes to *verbal* win > no-win trials by EFA.

	MNI	k	t-	p
Region	coordinates	cluster size	value	value*
Pallidum right	22 −8 −4	29	3.60	.006
Pallidum left	−24 −12 −4	15	3.41	.012
Posterior hippocampus right	28 −30 −10	61	4.12	.004
Insula left	−46 4 −4	54	4.44	.002
Insula right	48 8 −2	93	4.24	.004
Substantia nigra left	−12 −20 −10	15	3.59	.002
Substantia nigra right	10 −18 −12	14	3.02	.010

* FWE corrected at a threshold of .05, k≥10.

Furthermore, a significant negative correlation between neuronal activation during reward anticipation and delivery emerged, indicating that activation of the left VS decreased during anticipation when activation of the right insula [r = −.189; p = .016] and right pallidum [r = −.225; p = .004] increased during delivery (see Figure S2).

**3. Interaction between *Phase* (Anticipation/Delivery) and EFA.** A significant interaction between Phase and EFA was obtained in all three ROIs (left VS: F_(1,160)_ = 7.36, p = .007; right insula: F_(1,160)_ = 10.32, p = .002; left putamen: F_(1,160)_ = 10.75, p = .001), when considering the same ROIs for both stages of reward processing. Post-hoc regression analysis revealed that the interaction effect in the left VS was driven by anticipation alone (anticipation: F_(1,160)_ = 14.33, p<.001; delivery: F_(1,160)_ = 1.31, p = .255), while, in the other ROIs, it was driven by both anticipation and delivery (right insula: anticipation: F_(1,160)_ = 4.33, p = .039; delivery: F_(1,160)_ = 7.68, p = .006; left putamen: anticipation: F_(1,160)_ = 11.58, p = .001; delivery: F_(1,160)_ = 4.22, p = .042). Additionally, a significant interaction between Phase and EFA emerged, when considering different ROIs for both reward processing stages (anticipation: left VS; delivery: right insula; F_(1,160)_ = 17.72, p<.001).

### Correlation of fMRI activation with behavioral measures and CNV

Negative correlations between RT to monetary trials and neuronal activation (VS, putamen, thalamus) during reward anticipation, contrasting monetary over verbal reward, occurred in several regions (VS: r = −.347; p<.01; putamen: r = −.367; p<.01; thalamus: r = −.345; p = <.01). The analogous correlation emerged between RT and CNV contrasting monetary over verbal reward (r = −.215; p<.01). No significant correlation for RT of verbal trials was found. Furthermore, the number of monetary win trials was positively related to neuronal activation during reward anticipation contrasting monetary over verbal reward (VS: r = −.321; p<.01; putamen: r = −.360; p<.01; thalamus: r = −.328; p = <.01). A similar finding was obtained for the number of verbal win trials (VS: r = −.198; p<.05; putamen: r = −.220; p<.01; thalamus: r = −.187; p = <.05). There was no significant correlation between the number of win trials and CNV. A significant negative correlation of fMRI activation with the CNV during reward anticipation (contrasting monetary to verbal cues for both measures) emerged (VS: r = −.215; p = .006; putamen: r = −.222; p = .004; thalamus: r = −.250; p = .001), showing that the CNV decreased when fMRI activity increased.

### Correlation of neuronal activity (fMRI/CNV) and EFA with lifetime ADHD

There was a significant correlation of fMRI activation contrasting monetary to verbal cues during reward anticipation in the left VS with ADHD symptoms, revealing decreasing activity with the number of ADHD symptoms (r = −.160; p = .042). Moreover, a significant correlation of fMRI activation contrasting win to no-win trials during reward delivery of the right insula with ADHD symptoms was obtained, showing increasing activity with the number of ADHD symptoms (r = .203; p = .01). In contrast, the CNV was found to be unrelated to ADHD symptoms (r = .10; p = .207). Furthermore, EFA correlated significantly with ADHD symptoms (r = .285; p<.001). The mediation analysis of the association between EFA and ADHD symptoms revealed no significant mediation by neuronal activation (left VS: Z = 1.01, p = .31, 95% CI: −.019 □ .084; right insula: Z = 1.52, p = .13, 95% CI: 0 □ .105).

## Discussion

The current simultaneous EEG-fMRI study investigated the long-term impact of early life adversity on neuronal alterations of the reward system into adulthood. Using data of an epidemiological cohort study from birth onwards, the results presented above provided evidence of altered reward processing later in life following exposure to early adversity. Specifically, our findings demonstrated a differential impact of adversity on neural responding to distinct phases of reward processing, indicating that the activation of specific reward-related brain areas (VS, putamen, thalamus) decreased with the level of adversity during reward anticipation, while there was an increase in activity of other reward-related areas (pallidum, insula, substantia nigra, right posterior hippocampus) with the level of adversity during reward delivery. The fMRI finding during reward anticipation converged with EEG results showing a negative association between the CNV and adversity, matching the negative correlation of CNV with fMRI activation. Further analysis of the single reward conditions revealed striking effects of early adversity on the processing of verbal reward, which accounted for major parts of the total reward-related activity during the delivery phase.

### Reward anticipation & early life stress

The results of the present study replicate recent findings with regard to reward anticipation [10], [33], highlighting deficits in the reward processing circuitry associated with exposure to early adversity. While in these studies, small samples of individuals exposed to severe childhood adversity (maltreatment, deprivation) were investigated, the present study extends these findings to a substantially larger number of individuals from an epidemiological cohort study who experienced low to moderate levels of adversity. Moreover, in contrast to these studies, which included maltreated individuals with a current psychiatric disorder, the present analysis focused on currently healthy individuals only. The observed activation of the VS, the putamen and the thalamus is in accordance with previous research, supporting the assumption of a specific reward circuitry affected by stress in early life [11], [12]. Interestingly, while Dillon et al. [10] reported less activation for maltreated individuals in the left pallidum and putamen, we replicated this effect for the putamen and, additionally, for the thalamus and the VS, the latter serving as the core region of reward processing. The prominent role of the thalamus in the reward circuit has recently been established by the demonstration of a strong direct link to the nucleus accumbens in studies measuring effective connectivity using dynamic causal modeling [38], [66].

The finding of a negative association between the CNV and early adversity, which to our knowledge is new to the field, provides additional evidence to substantiate the hypothesis of an adversity-driven dysfunctional neuronal reward circuit, suggesting that reward processing is already impaired less than three seconds after cue onset. This result supports the notion of a reward-driven variability of the CNV-like activity preceding uncertain feedback, and is in accordance with previous findings of a relationship between CNV and reward anticipation [23]–[25]. Along the same lines, slower RTs with the level of EFA during reward anticipation were found to be linked with blunted neuronal activity and a reduced CNV.

### Reward delivery & early life stress

The finding that neural activity in reward-related areas increased with the level of early adversity during reward delivery is in contrast to previous studies [10], [33], which were unable to establish an effect of adversity on the processing of reward outcomes. Several reasons may account for this inconsistency: First, given our substantially larger sample, the present study had a considerably higher power to uncover effects of adversity. Second, a continuous, prospective measure of adversity such as applied in this study may enable the detection of subtle adversity-modulated reward activation in contrast to a case-control design. Third, differences in the MID tasks used to assess reward processing may contribute to the discrepant findings. While in our study, monetary reward was contrasted with verbal reward as a control condition, others included a loss condition or used different intensities of monetary reward as contrasts [10], [33]. This reduces the number of trials per condition and, in combination with small sample sizes, may lead to reduced effect sizes and less sensitivity to reward outcome.

The activation of the pallidum, insula, hippocampus and substantia nigra demonstrated here is in accordance with the assumption of the phasic transmission of reward information via dopaminergic projections from the midbrain to the VS [67]. The hippocampal area plays a prominent role in regulating the reward circuit [68], [69] by showing afferent and efferent projections to the VS [70], regulating emotional, motivational and learning processes [71], [72]. The observation of more pronounced EFA-related modulation following verbal reward may indicate a specific sensitivity for social reward appreciation in individuals exposed to high adversity in early childhood, which might be specifically represented by activation of the hippocampus. The VS directly projects to the pallidum, integrating reward information and driving action output [11], [73]. Moreover, pallidum activation affected by early adversity, as previously found for reward anticipation [10], suggests a high involvement of the basal ganglia in reward processing, including both the anticipation and outcome phase. A specific activation of the medial orbitofrontal and ventromedial prefrontal cortex during reward delivery as proposed by Diekhof et al. [11] was not supported by the current study. This might be due to the absence of different magnitudes in monetary rewards in the present MID paradigm, which have been suggested to be processed by frontal activation.

Our finding that the impact of early adversity on reward outcome was only marked in the verbal reward control condition highlights the special reward quality of this condition, and may suggest that individuals exposed to early adversity are particularly prone to social rewards, such as verbal praise. This higher responsiveness to social rewards may result from the experience of poor parenting during childhood in individuals exposed to early family adversity [74]–[76], which may have increased the rewarding effect of social stimuli later in life. A retrospective cohort study by Baker and Hoerger [77] has implicated dysfunctional parenting, including low parental warmth or high rejection and control, in the development of difficulty delaying gratification. Such findings may support the hypothesis that poor parenting may lead to a reward deficiency syndrome [78], resulting in increased social reward retrieval.

### Reward processing & acute vs. early life stress

In contrast to our results, Kumar et al. [34], when investigating the impact of acute stress on reward processing, reported an opposite activation pattern in regions (caudate [VS], putamen) partly overlapping with ours, indicating increased neuronal activation during reward anticipation and decreased activation while receiving a reward. These findings underpin the functional differences between the impact of acute stress vs. early life stress on reward processing. Specifically, it has been demonstrated that the stress (HPA axis) and the reward system show considerable overlap on both the structural and the functional level [35]. Acute stress leads to an up regulation of HPA axis activity, thereby increasing motivation and approach behaviors, but blunting reward responsiveness [79]–[82]. In contrast, early life stress may result in an adaptation of HPA axis activity with first increased cortisol release during stress exposure followed by later hypocortisolism [83], [84] which might be linked to decreased dopamine transmission during reward anticipation but increased when receiving a reward.

### Reward processing, early life stress & ADHD

Our findings provide additional insights into the relationship between altered reward processing in individuals exposed to early adversity and mental disorders related to dysfunction of the dopamine reward circuit, such as ADHD. The differential effect of early life stress on both stages of reward processing, characterized by hyporesponsiveness in individuals exposed to high early adversity when anticipating a monetary reward and hyperresponsiveness when receiving a reward, is in line with the literature on dysfunctional reward processing in ADHD [8], [9], [14]–[17]. Accordingly, there was a significant association between fMRI activation in reward-related regions and lifetime ADHD symptoms, showing the same differential effect on anticipatory and delivery phases as observed for early life stress. Given that the latter represents a major risk factor of ADHD [43], this might suggest an impact of EFA on ADHD via a dysfunctional reward pathway. Here, this pathway could not be confirmed by the mediation analysis. In contrast to fMRI, the CNV proved to be unrelated to ADHD. As the CNV has been identified as a stable ADHD marker for preparation deficits measured by cognitive paradigms such as the CPT [29], [30], this result might be an effect of paradigm. Although there was a significant impact of EFA on the CNV, reward anticipation as part of an emotional paradigm might be less sensitive to ADHD effects than cognitive paradigms. However, given the significant correlation between CNV and fMRI activation, the different measures alone could not explain this differential effect.

### Limitations

Several limitations have to be considered in the interpretation of our results. First, due to reduced data quality following the button press, it was not possible to analyze EEG feedback components such as the feedback-related negativity [26]. However, it would be most interesting to examine whether EEG feedback components would display a similar pattern of outcome-related EFA effects to that found for fMRI. Second, given the small effect size of EFA and the fact that several characteristics of EFA would not change during the individual's life course, the results cannot be attributed to early life stress alone but probably also reflects stress during later development [2]. Third, the present results do not provide evidence of the mechanisms mediating between exposure to EFA and altered reward processing in adulthood. Several mechanisms have been discussed as determining the transduction of environmental influences into changes in brain physiology and morphology. Among these, a major role has been attributed to epigenetic regulation [85], [86]. Hence, the investigation of epigenetic signatures induced by exposure to EFA that persist into adulthood appears to be a promising research perspective. Fourth, current research has highlighted the differential susceptibility of individuals to EFA. Greater insight into the interplay between environmental and genetic factors that affect reward processing may further contribute to a better understanding of the underlying mechanisms. Genes that have been shown to exert remarkable effects on the reward circuit include, among others, the dopamine transporter gene (DAT) [87], [88] and the catechol-O-methyltransferase gene (COMT) [6], [89], [90].

## Conclusion

In sum, the present findings provide evidence of a differential long-term impact of early life adversity on two distinct phases of reward processing in adulthood, characterized by hyporesponsiveness during reward anticipation followed by hyperresponsiveness when receiving a reward. Moreover, a similar activation pattern related to lifetime ADHD may suggest that the impact of early life stress on ADHD may possibly be mediated by a dysfunctional reward pathway.

## Supporting Information

Figure S1 **Whole-brain task effects a) during the anticipation of monetary vs. verbal rewards, indicating significantly higher activation in the ventral striatum (VS), thalamus, anterior cingulate cortex, supplementary motor area, primary motor area and occipital cortex and b) during reward delivery (win vs. no-win), yielding significantly higher activation in the putamen, caudate, left inferior frontal gyrus, right dorsolateral prefrontal cortex, primary motor area, right medial frontal gyrus and occipital cortex (all p_FWE_<.0001; k≥20).**
(TIF)

Figure S2 **Significant negative correlation of activation in the left VS during reward anticipation with a) right insula activation (pooled reward) [r = −.189; p = .016] and b) right pallidum activation (verbal reward) [r = −.225; p = .004] during reward delivery.**
(TIF)

Figure S3 **Grand average ERPs showing the stronger contingent negative variation (CNV) developing at electrode Cz (marked with an asterisk) after the presentation of monetary (happy smiley, black curve) compared to verbal (scrambled smiley, red curve) reward cues; p<.001 in the analysis time window (blue, 2–3 sec following cue onset and preceding target onset on all trials).**
(TIF)

Table S1 **Definition of early family adversity (EFA) items.**
(DOCX)
