# Correction: Impact of Early Life Adversity on Reward Processing in Young Adults: EEG-fMRI Results from a Prospective Study over 25 Years

**DOI:** 10.1371/journal.pone.0112155

**Published:** 2014-10-24

**Authors:** The PLOS ONE Staff

There are errors in the author affiliations. The correct affiliations should appear as shown here:

Regina Boecker^1^, Nathalie E. Holz^1^, Arlette F. Buchmann^1^, Dorothea Blomeyer^1^, Michael M. Plichta^2^, Isabella Wolf^1,3^, Sarah Baumeister^1^, Andreas Meyer-Lindenberg^2^, Tobias Banaschewski^1^, Daniel Brandeis^1,4,5,6☯^, Manfred Laucht^1,7☯^*

1 Department of Child and Adolescent Psychiatry and Psychotherapy, Central Institute of Mental Health Medical Faculty Mannheim/Heidelberg University, Mannheim, Germany,

2 Department of Psychiatry and Psychotherapy, Central Institute of Mental Health Medical Faculty Mannheim/Heidelberg University, Mannheim, Germany,

3 Department of Neuroimaging, Central Institute of Mental Health Medical Faculty Mannheim/Heidelberg University, Mannheim, Germany,

4 Department of Child and Adolescent Psychiatry, University of Zurich, Zurich, Switzerland,

5 Center for Integrative Human Physiology, University of Zurich, Zurich, Switzerland,

6 Neuroscience Center Zurich, University of Zurich and ETH Zurich, Zurich, Switzerland,

7 Department of Psychology, University of Potsdam, Potsdam, Germany

* Email: manfred.laucht@zi-mannheim.de

☯ These authors contributed equally to this work.
