# Supplementary figures and images for: Impact of Early Life Adversity on Reward Processing in Young Adults: EEG-fMRI Results from a Prospective Study over 25 Years
Source: PLoS One. 2014 Aug 13;9(8):e104185. doi: 10.1371/journal.pone.0104185 (PMC4131910; doi:10.1371/journal.pone.0104185)

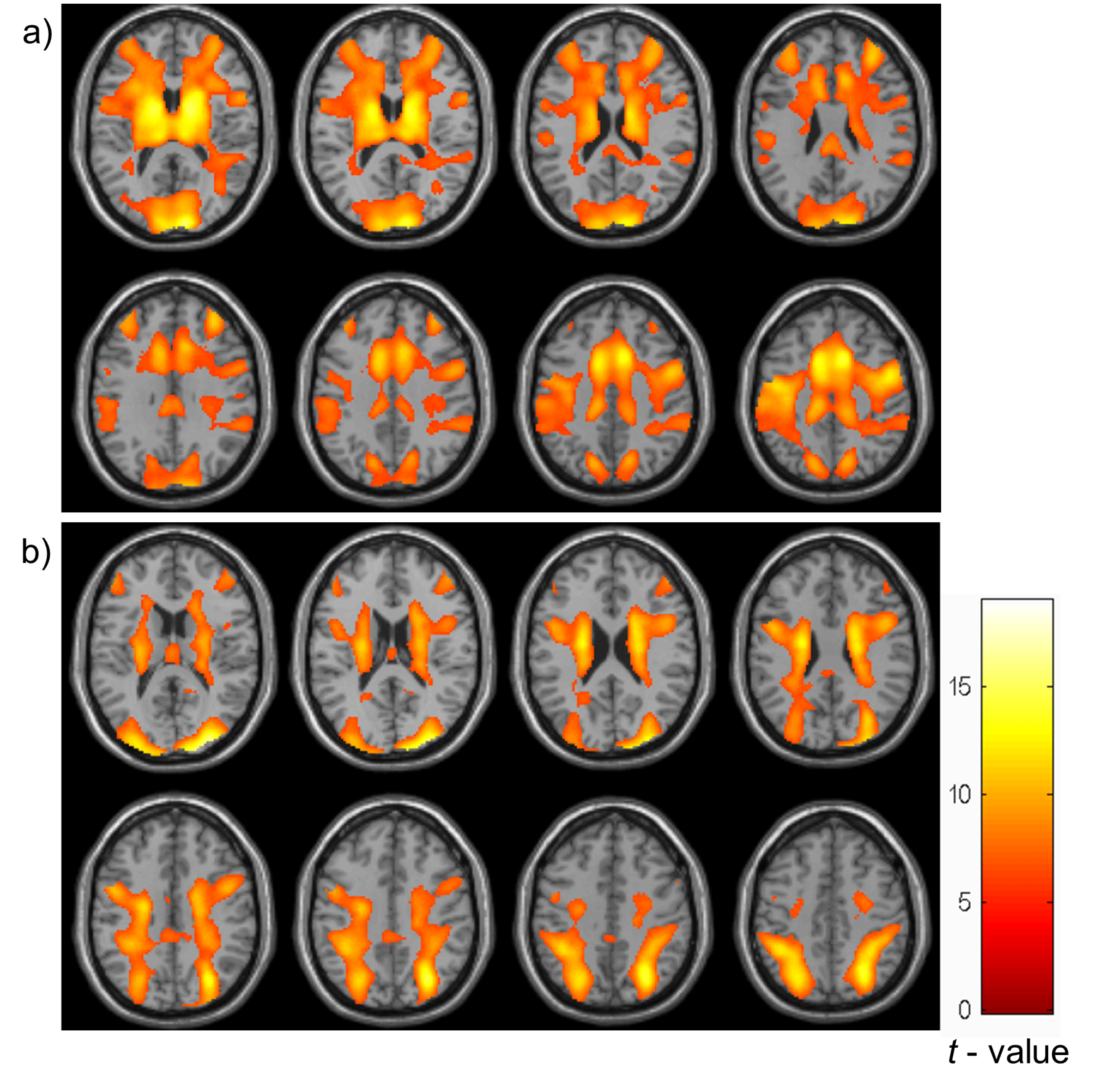

Supplement: Figure S1 — Whole-brain task effects a) during the anticipation of monetary vs. verbal rewards, indicating significantly higher activation in the ventral striatum (VS), thalamus, anterior cingulate cortex, supplementary motor area, primary motor area and occipital cortex and b) during reward delivery (win vs. no-win), yielding significantly higher activation in the putamen, caudate, left inferior frontal gyrus, right dorsolateral prefrontal cortex, primary motor area, right medial frontal gyrus and occipital cortex (all pFWE<.0001; k≥20). (TIF) [file pone.0104185.s001.tif]

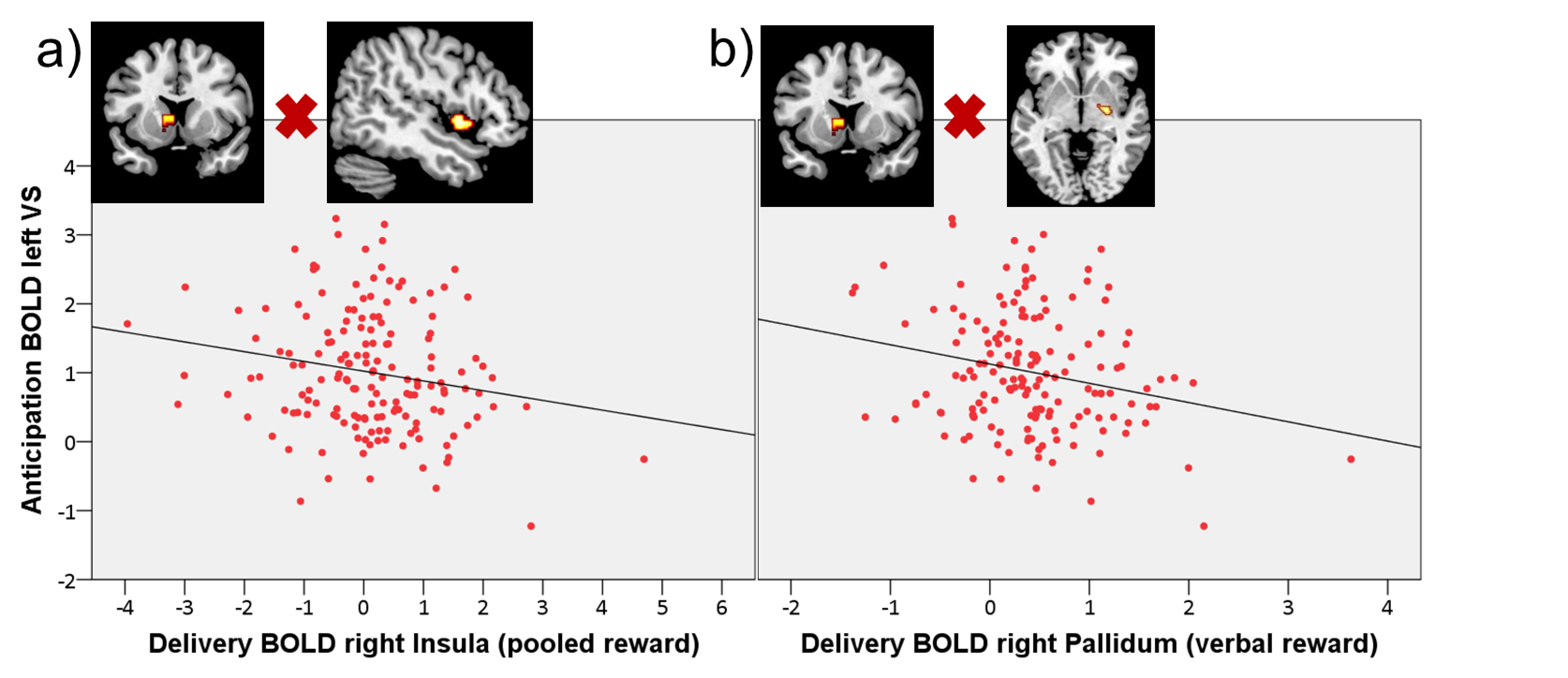

Supplement: Figure S2 — Significant negative correlation of activation in the left VS during reward anticipation with a) right insula activation (pooled reward) [r = −.189; p = .016] and b) right pallidum activation (verbal reward) [r = −.225; p = .004] during reward delivery. (TIF) [file pone.0104185.s002.tif]

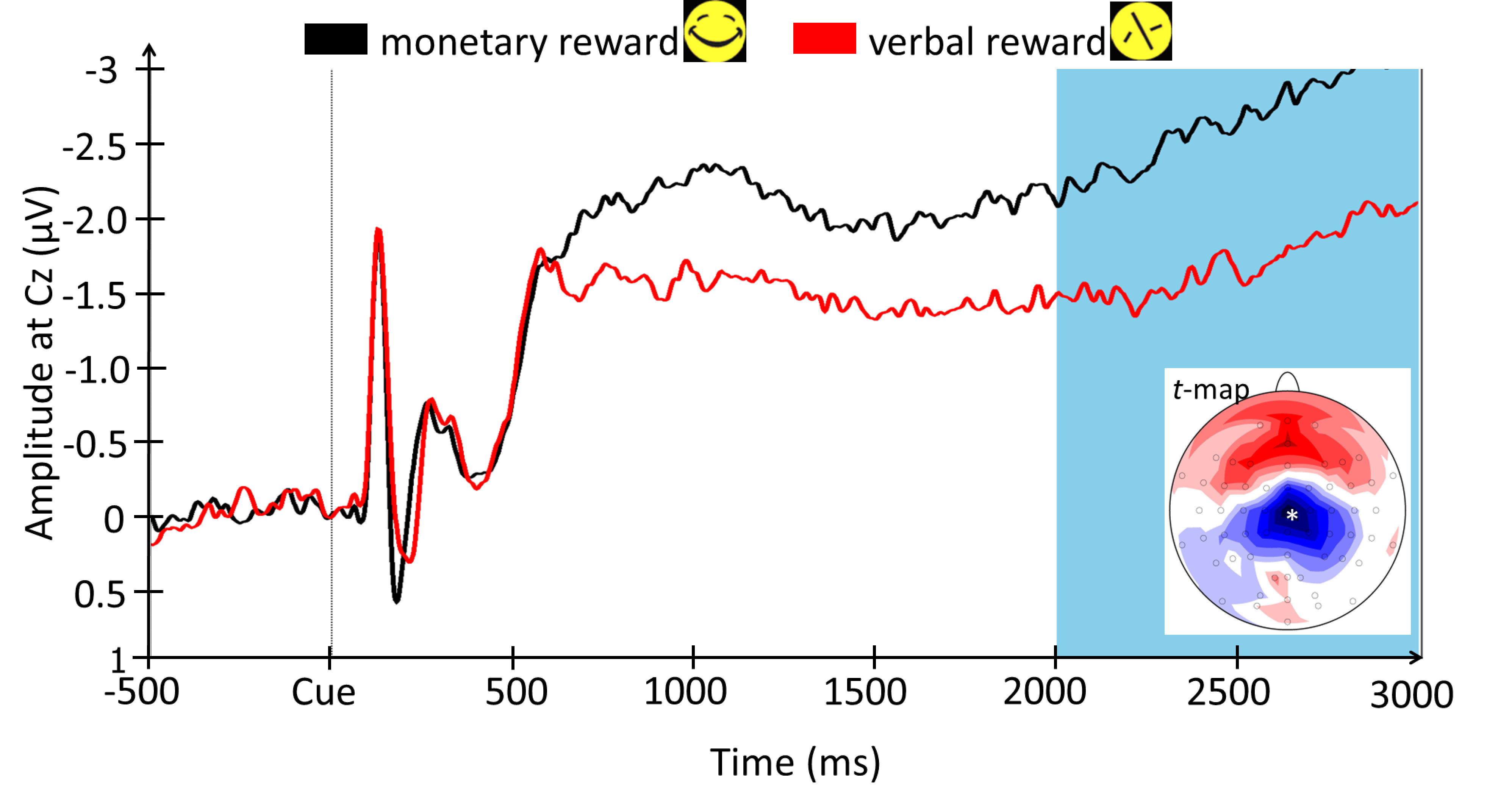

Supplement: Figure S3 — Grand average ERPs showing the stronger contingent negative variation (CNV) developing at electrode Cz (marked with an asterisk) after the presentation of monetary (happy smiley, black curve) compared to verbal (scrambled smiley, red curve) reward cues; p<.001 in the analysis time window (blue, 2–3 sec following cue onset and preceding target onset on all trials). (TIF) [file pone.0104185.s003.tif]
