# Supplementary material for: Impact of Early Life Adversity on Reward Processing in Young Adults: EEG-fMRI Results from a Prospective Study over 25 Years
Source: PLoS One. 2014 Aug 13;9(8):e104185. doi: 10.1371/journal.pone.0104185 (PMC4131910; doi:10.1371/journal.pone.0104185)
Supplement: Table S1 — Definition of early family adversity (EFA) items. (DOCX) [file pone.0104185.s004.docx]

Supplementary Table S1: Definition of early family adversity (EFA) items

| **Item** | **Definition** |
| --- | --- |
| 1. **Low educational level** | Parent without completed school education or without skilled job training |
| 1. **Overcrowding** | More than 1.0 person per room or size of housing ≤50 m^2^ |
| 1. **Parental psychiatric disorder** | Moderate to severe disorder according to DSM-III-R criteria |
| 1. **History of parental broken home or delinquency** | Institutional care of a parent/more than two changes of parental figures until the age of 18 or history of parental delinquency |
| 1. **Marital discord** | Low quality of partnership in two out of three areas (harmony, communication, emotional warmth) |
| 1. **Early parenthood** | Age of a parent ≤18 years at child birth or relationship between parents lasting less than 6 months at time of conception |
| 1. **One-parent family** | At child birth |
| 1. **Unwanted pregnancy** | An abortion was seriously considered |
| 1. **Poor social integration and support of parents** | Lack of friends and lack of help in child care |
| 1. **Severe chronic difficulties** | Affecting a parent lasting more than one year |
| 1. **Poor coping skills of a parent** | Inadequate coping with stressful events of the past year |
